# Full Mouth Oral Rehabilitation by Maxillary Implant Supported Hybrid Denture Employing a Fiber Reinforced Material Instead of Conventional PMMA

**DOI:** 10.1155/2015/841745

**Published:** 2015-10-18

**Authors:** Ala Hassan A. Qamheya, Sinem Yeniyol, Volkan Arısan

**Affiliations:** Department of Oral Implantology, Faculty of Dentistry, Istanbul University, 34093 Istanbul, Turkey

## Abstract

Many people have life-long problems with their dentures, such as difficulties with speaking and eating, loose denture, and sore mouth syndrome. The evolution of dental implant supported prosthesis gives these patients normal healthy life for their functional and esthetic advantages. This case report presents the fabrication of maxillary implant supported hybrid prosthesis by using Nanofilled Composite (NFC) material in teeth construction to rehabilitate a complete denture wearer patient.

## 1. Introduction

In contrast to the focus on achieving successful osseointegration of dental implants, nowadays obtaining the most natural-looking smiles through preserving the anatomy of the soft tissues or creating them either by tissue regeneration or prosthetic materials is the main concerns of the practitioners [1].

Elderly patients visit the dental clinics seeking for a good smile which was lost due to the loss of the teeth, supporting alveolar bone, and muscles. To restore these units, complete edentulous patients must be treated by dental implants [2]. Treatment options may range from the use of removable implant supported dentures to the creation of fixed implant supported restorations. This treatment choice depends on the patient's anatomical limitations and personal preferences, including acceptance of extensive surgical procedures to restore the bone and/or soft tissue [1].

Implant supported overdentures and hybrid prosthesis often provide support for the soft tissues of the face when compared to the traditional fixed prosthesis. With the emergence of computer-aided designs and the development of prosthetic materials, soft tissue loss can be easily replaced and even pink interdental papilla can be artificially created [3].

When adequate number of implants is present in an arch, a traditional fixed bridge is the prosthetic modality of choice. Often this is not an option in the maxilla due to combined vertical and horizontal resorption of bone and tilted positions of the implants. In this instance, a traditional fixed bridge would not meet the patient's requirements for hygiene maintenance, esthetics, phonetics, and comfort. In addition to that, pink porcelain is less natural-looking and it usually requires more baking cycles that increase the risk of porcelain fracture [2]. These complications can be solved by fabricating hybrid prosthesis that can easily replace the soft tissue, and, concerning their shock absorbing properties, it can reduce the mechanical and biological problems such as component fracture, screw loosening, and bone resorption [4].

With regard to the rapid wear of acrylic denture teeth as well as the stress generation of porcelain teeth within the framework leading up to marginal bone around dental implants, use of veneer materials has been widely accepted in implant dentistry for their stress absorption and less wear characteristics [5, 6].

This clinical report presents the fabrication of maxillary implant supported hybrid prosthesis and the Nanofilled Composite (NFC) material used for its construction.

## 2. Diagnosis and Treatment Planning

**Figure 1:**
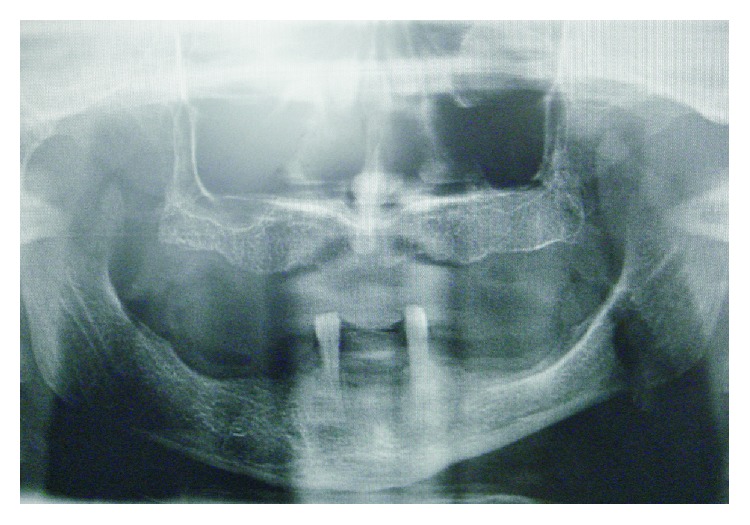
Panoramic radiograph of patient with two poor-prognosed mandibular teeth.

A 55-year-old female applied to our clinic (Department of Oral Implantology, Faculty of Dentistry, Istanbul University) seeking for full prosthetic oral rehabilitation. Patient had already been a wearer of complete dentures in both arches and she wanted them to be replaced by fixed prostheses. After clinical and radiological assessments (Figure 1), considering the loss of the bone and labial support, two treatment options had been presented for the patient: implant supported overdentures or implant supported hybrid dentures. The former was refused by the patient. Considering her financial status, lower arch was preferred to be treated with an implant supported overdenture, considering the insufficient bone height and width.

## 3. Surgical Stage

**Figure 2:**
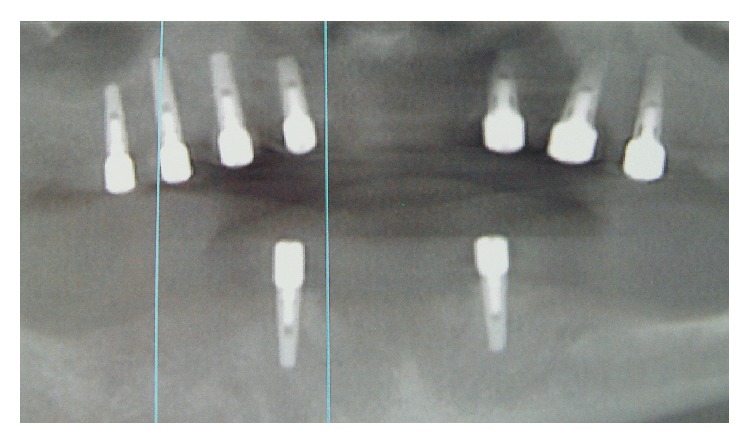
Panoramic radiograph after implant insertion.

**Figure 3:**
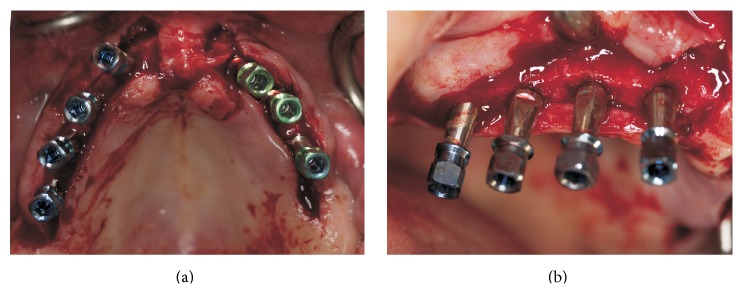
(a and b) Clinical view of the implants after surgical insertion.

**Figure 4:**
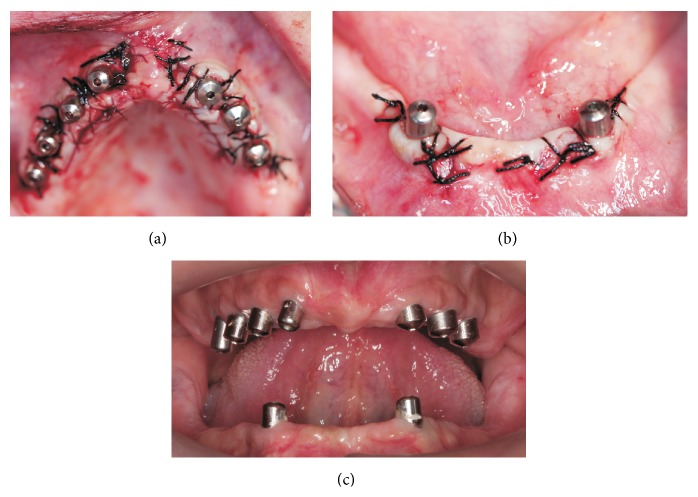
(a and b) Gingival formers were fastened and the flaps were repositioned with interrupted 3.0 silk sutures. (c) The healing was uneventful for the following ten days.

After clinical and radiological assessments including computerized tomography, it was decided that the patient was a candidate to receive implant supported rehabilitation. Maxillary and mandibular restorations were planned to restore both hard and soft tissues. Thus, seven internal hexed titanium implants (WIS, Merate, Italy) were inserted according to manufacturer's recommendations. Implant locations were 15, 13, 11, 16, 26, 23, and 21 (Figures 2 and 3). Two-staged approach was employed and implants were left to submerged healing. After four months of an osseointegration period, healing abutments were placed by a palatal crestal incision. A coronally repositioned flap from the palatal soft tissue was achieved in order to facilitate a gain of attached tissue and tension-free closure of the flap around the healing abutments (Figure 4).

## 4. Prosthetic Stage

**Figure 5:**
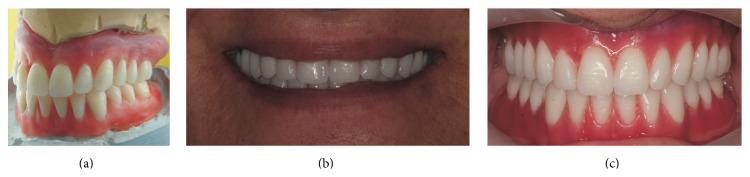
(a, b, and c) Wax-up try-in for determining intermaxillary vertical dimensions and the support.

Soft tissue around the healing abutments was left to mature for 3 weeks after the second-stage surgery. Impressions were taken using the pick-up technique and a try-in for the quantitative assessment of occlusal vertical dimension and aesthetic appearance evaluation was performed on the wax-up. To determine the position of the future teeth, a complete denture try-in with plastic teeth was made. This setup is considered as the facial border of the future planned wax-up and it also helps the technician to construct a computerized design for this hybrid prosthesis which stimulates the final restoration (Figure 5).

**Figure 6:**
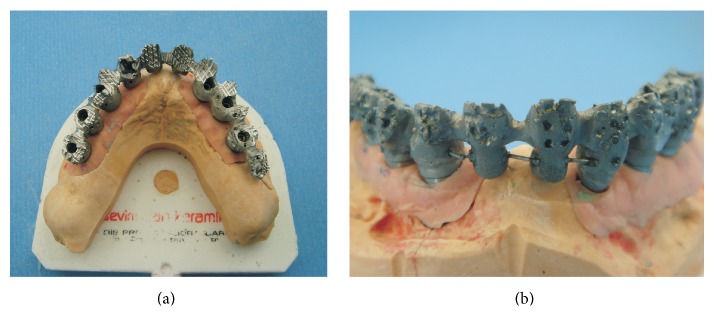
(a) Occlusal and (b) frontal view of laser sintered metal substructure.

**Figure 7:**
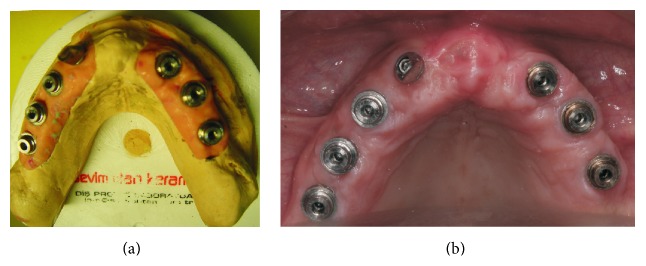
Occlusal view of the abutments (a) on the study cast and (b) intraorally.

**Figure 8:**
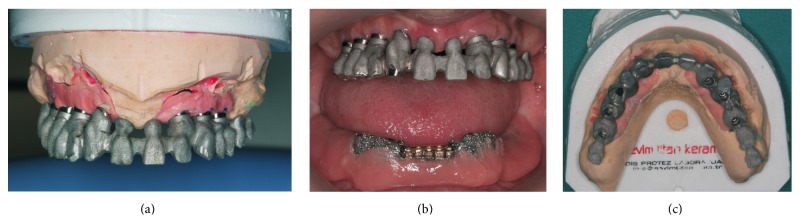
(a) Occlusal view of metal substructure, (b) frontal view of metal substructure, and (c) metal try-in.

A porcelain metal substructure was fabricated by CAD-CAM technology, and this structure was sintered by laser to create retention grooves that increase the mechanical retention between the metal and the composite material. In addition to the mechanical retention, chemical retention was provided by a proprietary bonding agent (GC Metalprimer II) (Figures 6, 7, and 8) [7].

**Figure 9:**
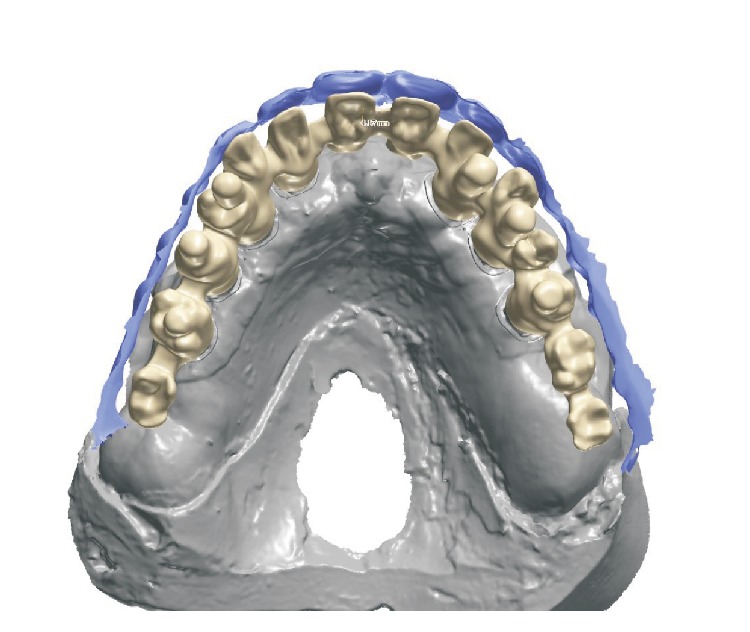
Computerized view of model and metal substructure.

Hybrid prosthesis was fixed to the implant abutments at sites 15, 11, 16, 26, 23, and 21 by occlusal screws. Implant at site 13 was found to be placed, tilted to the vestibular aspect during the first surgery, so this abutment at site 13 was cemented to the framework to provide a parallel path of insertion to the framework and to improve this implant's emergence profile (Figure 9).

**Figure 10:**
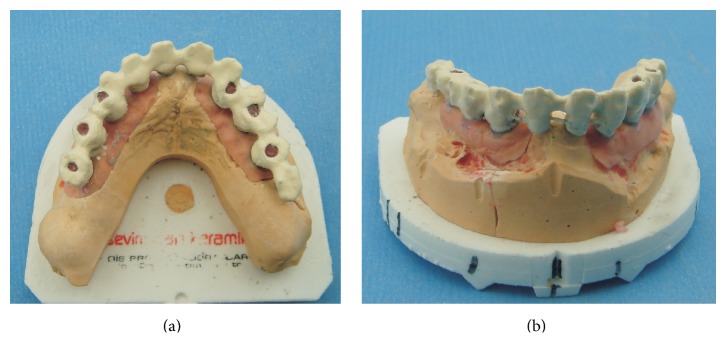
(a) Occlusal and (b) frontal view of the first composite layer that is bonded to the metal substructure.

**Figure 11:**
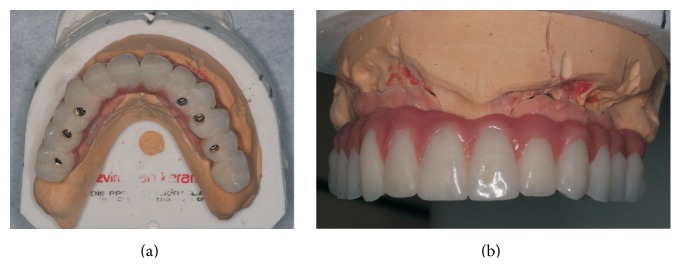
(a) Frontal and (b) occlusal view for hybrid prosthesis.

**Figure 12:**
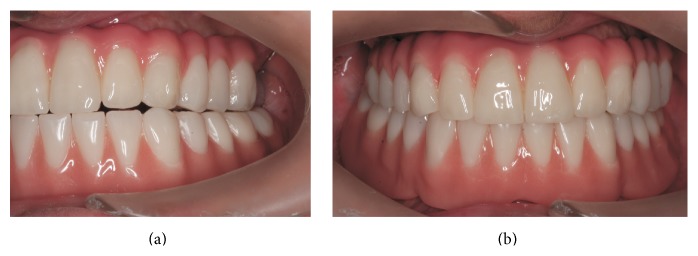
Occlusal adjustments at (a) lateral movement and (b) centric occlusion.

Hybrid denture teeth and the artificial soft tissue were made by 75% Nanofilled Composite and 25% acryl in composition (NFC; UDMA with inorganic nanofillers, Condulor). The lower overdenture was made by acrylic teeth and metal supported acrylic flange. The abutments were screwed to the implants. Hybrid denture was cemented to number 13 and screwed to the other abutments though a torque wrench to generate the preload that keeps the prosthetic components together [8]. After that occlusion was adjusted, the end result provided adequate contour to facilitate maintenance and healthy gingival tissues (Figures 10, 11, and 12).

## 5. Discussion

Fabrication of hybrid dentures, in patients with excessive interocclusal space, provides the dentist with several advantages for the esthetic appearance. Replacement and soft tissue support decrease in the bulkiness of metal substructure and in the height of crowns compared to the metal supported porcelain prosthesis. In addition to these esthetics advantages, hybrid prosthesis works as shock absorbent and force distributer reducing the sudden load forces on implants. On the other hand, prosthetic parts replacing the soft tissue enhance oral hygiene by the food being swiped away as a self-cleansing enhancement.

Currently, instead of using acrylic or porcelain artificial teeth, new restorative materials like poly methyl methacrylate (PMMA) and urethane dimethacrylate (UDMA) containing composite materials are developed for the fabrication of implant supported prosthesis [5]. NFC material was stated to show significantly less wear than DCL (double cross-linked PMMA). Since UDMA-containing composites are more wear resistant than PMMA, UDMA-containing composites (NFC^+^, Candulor, Switzerland) are used in our case that consisted of highly dispersed silicone dioxide and highly cross-linked UDMA matrix [6]. For the mechanical properties gained by adding cross-linked materials, in case of NFC, nanosized inorganic fillers give the homogeneity of the material [8].

Hardness and wear resistance of nanocomposite denture teeth, microfilled composite teeth, cross-linked denture teeth, and the commonly used acrylic denture teeth were evaluated. The resistance of the nanocomposite denture teeth was found more than acrylic resin but less than microfilled composite denture teeth [8].

Hirano et al. stated that there are significant differences (*p* < 0.05) in the wear rates between denture teeth composite made and acrylic made after 5000 and 10000 cycles, where composite teeth had less wear rate than the acrylic teeth [9].

Hahnel et al. stated that the wear resistance depends on the type of antagonist teeth, as condyloform II NFC presented significantly less vertical substance loss than (DCL) double cross linked PMMA. At the same way when ceramic was used as antagonist, the condyloform II NFC had significantly less vertical loss than DCL but more wear and volume loss than when artificial resin antagonist teeth were used [10].

The factors that should be taken into consideration to decrease stress upon the hybrid prosthesis' components are the correct arrangement of implants, elimination of cantilevers, fine occlusal adjustment, fabrication of a stiff framework, and use of stress absorbing materials [3, 11]. Some mechanical and biological complications can be faced due to the generation of high stresses which result in screw loosening and component fracture, biological bone resorption, and detachment of veneering material from its framework [2, 3]. But when multiple materials are used to fabricate implant supported prosthesis, they can decrease these complications since they work as a force distributer and absorber [7].

## 6. Conclusion

Implant supported prosthetic rehabilitation of the patients with severe alveolar bone loss can be facilitated with the use of Nanofilled Composite (NFC) material reinforced hybrid dentures fabricated by CAD/CAM technique.

## References

[1] Steigmann M. (2008). Aesthetic flap design for correction of buccal fenestration defects. *Practical Procedures & Aesthetic Dentistry: PPAD*.

[2] Montero J., de Paula C. M., Albaladejo A. (2012). The ‘Toronto prosthesis’, an appealing method for restoring patients candidates for hybrid overdentures: a case report. *Journal of Clinical and Experimental Dentistry*.

[3] Gonzalez J. (2014). The evolution of dental materials for hybrid prosthesis. *Open Dentistry Journal*.

[4] Kwon T., Bain P. A., Levin L. (2014). Systematic review of short- (5–10 years) and long-term (10 years or more) survival and success of full-arch fixed dental hybrid prostheses and supporting implants. *Journal of Dentistry*.

[5] Heintze S. D., Zellweger G., Grunert I., Muñoz-Viveros C. A., Hagenbuch K. (2012). Laboratory methods for evaluating the wear of denture teeth and their correlation with clinical results. *Dental Materials*.

[6] Radhi A., Juszczyk A. S., Curtis R. V., Sherriff M., Radford D. R., Clark R. K. F. (2008). Effect of GC METALPRIMER II on bond strength of heat-cured acrylic resin to titanium alloy (Ti-6Al-4V) with two different surface treatments. *The European Journal of Prosthodontics and Restorative Dentistry*.

[7] Silva G. C., Cornacchia T. M., De Magalhães C. S., Bueno A. C., Moreira A. N. (2014). Biomechanical evaluation of screw- and cement-retained implant-supported prostheses: a nonlinear finite element analysis. *Journal of Prosthetic Dentistry*.

[8] Drago C., Howell K. (2012). Concepts for designing and fabricating metal implant frameworks for hybrid implant prostheses. *Journal of Prosthodontics*.

[9] Suzuki S. (2004). In vitro wear of nano-composite denture teeth. *Journal of Prosthodontics*.

[10] Hirano S., May K. B., Wagner W. C., Hacker C. H. (1998). In vitro wear of resin denture teeth. *The Journal of Prosthetic Dentistry*.

[11] Hahnel S., Behr M., Handel G., Rosentritt M. (2009). Two-body wear of artificial acrylic and composite resin teeth in relation to antagonist material. *Journal of Prosthetic Dentistry*.

